# Characterization of the *OFP* Gene Family and its Putative Involvement of Tuberous Root Shape in Radish

**DOI:** 10.3390/ijms21041293

**Published:** 2020-02-14

**Authors:** Yanping Wang, Qingbiao Wang, Wei Hao, Honghe Sun, Li Zhang

**Affiliations:** 1 National Engineering Research Center for Vegetables, Beijing Academy of Agriculture and Forestry Sciences, Beijing 100097, China; wangyanping@nercv.org (Y.W.); wangqingbiao@nercv.org (Q.W.); 15076205291@163.com (W.H.); sunhonghe@nercv.org (H.S.); 2 Key Laboratory of Biology and Genetic Improvement of Horticultural Crops (North China), Ministry of Agriculture and Rural Affairs of the People’s Republic of China, Beijing 100097, China; 3 Beijing Key Laboratory of Vegetable Germplasm Improvement, Beijing 100097, China

**Keywords:** ovate family protein, radish, tuberous root, root shape

## Abstract

The shape of the tuberous root, a very important quality trait, varies dramatically among radish cultivars. Ovate family proteins (*OFPs*) are plant-specific proteins that regulate multiple aspects of plant growth and development. To investigate the possible role of *OFPs* in radish tuberous root formation, 35 putative *RsOFPs* were identified from radish, and their expression patterns were detected during tuberous root development in six different radish cultivars. Phylogenetically, *RsOFP2.3* clustered together with *AtOFP1* and other members of this family that are known to regulate organ shape. Moreover, *RsOFP2.3* expression was negatively correlated with tuberous root elongation after the cortex splitting stage, which made this gene the top candidate for the involvement of tuberous root shape. To further characterize the function of *RsOFP2.3*, it was ectopically expressed in Arabidopsis. *RsOFP2.3* overexpression in Arabidopsis led to multiple phenotypical changes, especially the decreased length and increased width of the hypocotyl. Furthermore, *RsOFP2.3* expression was induced by all the five classic plant hormones except ethylene, and it was most sensitive to exogenous gibberellic acid treatment. We also found that *RsOFP2.3* was localized in the cytoplasm. Taken together, our results suggested the possible involvement for *RsOFP2.3* in suppressing radish tuberous root elongation and that it encodes a functional protein which mainly inhibits the elongation of Arabidopsis aerial organs.

## 1. Introduction

Radish (*Raphanus sativus* L.), belonging to the Brassicaceae family, is an important vegetable mainly consumed for its tuberous root [1]. During its long history of domestication, huge changes have occurred in radish roots. Wild radishes native to coastal areas of the Mediterranean Sea have a thin non-fleshy root, while modern cultivated radishes have edible enlarged roots that vary largely in size and shape [2]. In the west, radish is a small root vegetable grown within one month and usually used in salads. However, in East Asia, where the largest producers and consumers of radish are located, radish cultivars with large roots of various shapes are more popular and widely produced [3]. Root shape can be characterized using such descriptions as round, round-cylindrical, long-cylindrical, thick-cone, thin-cone, long-cone, and so on [4]. Examples of different root morphologies can be found among cultivated radishes, from very small (10 g per root) to giant (30 kg per root), and from oblate (root shape index < 1) to extremely elongated (root shape index > 100) [3]. Root shape and size are important traits that can influence the commercial quality of radishes, such as transportation efficiency, processing methods, and consumer appeal. Studies on radish tuberous root formation have been a hot topic recently, and have mainly focused on finding QTLs, differentially expressed genes or proteins related to storage root formation [5,6,7,8,9,10,11]. However, the developmental mechanisms underlying the beneficial alleles controlling root formation have not yet been fully elucidated. Recently, ovate family proteins (*OFPs*) have been implicated in the regulation of multiple aspects of plant development. *OFPs* are plant-specific regulators that derive their name from the OVATE domain, also known as DUF623 (Domain of Unknown Function 623), and encode proteins with a conserved 70 amino acid C-terminal domain [12,13,14,15]. *OFPs* are widely distributed and conserved across various plant species, such as Arabidopsis, rice, tomato, potato, melon, banana, and grape, and regulate multiple aspects of plant growth and development [16,17,18,19,20,21]. Overexpressing *AtOFPs* in Arabidopsis had pleiotropic effects, including kidney-shaped cotyledons, altered leaf phenotypes, and blunt-end siliques [14]. Furthermore, overexpression of several rice *OFPs* resulted in similar phenotypes to those seen in Arabidopsis, such as reduced height, altered leaf morphology, and seed shape [17]. It was also reported that *OsOFP6* regulates plant development and confers resistance to drought and cold stresses [22]. A recently published paper reported that tomato plants overexpressing *SlOFP20* displayed several phenotypic defects, including an altered floral architecture and reduced male fertility [23].

Another prominent role of *OFPs* is in the regulation of fruit shape. In tomato, a naturally occurring premature stop codon in *OVATE* affects fruit shape in a variety of ways depending on the genetic background, resulting in elongated fruits, pear-shaped, or round-shaped fruits [24,25,26]. Furthermore, *SlOFP20* was also found to contribute to natural fruit shape variation. Overexpression of *SlOFP20* in a pear-shaped tomato variety Yellow Year (*Solanum lycopersicum* L.) produced much rounder fruit; correspondingly, downregulation of *SlOFP20* in the close wild relative of tomato *S. pimpinellifolium* LA1589 (round-shaped) led to a more elongated fruit shape but only in the *ovate* background [21]. It was recently revealed that OVATE and *SlOFP20* genetically and physically interact with members of the TONNEAU1-recruitment motif (TRM) family proteins containing the M8 motif [21]. TRMs function in assembling the TTP (TON1-TRM-PP2A) complex, which is postulated to regulate the organization of microtubules arrays, and thus control cell division patterns and cell growth, and overall organ shape [27,28,29]. *OFPs* have also been found to play a role in the regulation of organ shape during development in many plant species. In melon, *CmOFP1a* is a candidate for the fruit size/fruit shape QTL *CmFS8.3/CmFSI8.3* [21]. In potato, fine mapping with a diploid potato F_2_ population derived from a cross between the round tuber parent M6 and the parent producing elongated tubers DM1–3 revealed the tuber shape QTL *Ro* was controlled by *StOFP20* [21]. In addition, *CsOFP1a* and *ClOFP1a* are clustered in the same clade as tomato *SlOFP20*. Thus, *CsOFP1a* and *ClOFP1a* are considered top candidates for the two QTLs in cucumber and watermelon, respectively [30]. However, whether *OFPs* in radish play a role in tuberous root shape formation remains unclear.

The release of *R. sativus* genome sequences by three independent groups has greatly facilitated gene family studies in radish [9,31,32]. In this study, 35 putative *RsOFPs* were isolated from these three radish genome databases by blast searching. The gene structures, phylogenetic/homologous relationships, and expression of the *RsOFP* genes were analyzed. Furthermore, the growth parameters of six radish cultivars featuring different tuberous root sizes and shapes were investigated, and the possible function of *RsOFP2.3*, the gene most likely related to tuberous root shape formation, was verified in Arabidopsis by ectopic expression. Its subcellular localization and responses to five classic phytohormones were also investigated. These results provide insights into the molecular mechanisms of tuberous root shape formation in radish and the roles of *OFP* genes in organ shape formation.

## 2. Results

### 2.1. Characterization of Radish OFP Genes

**Figure 1:**
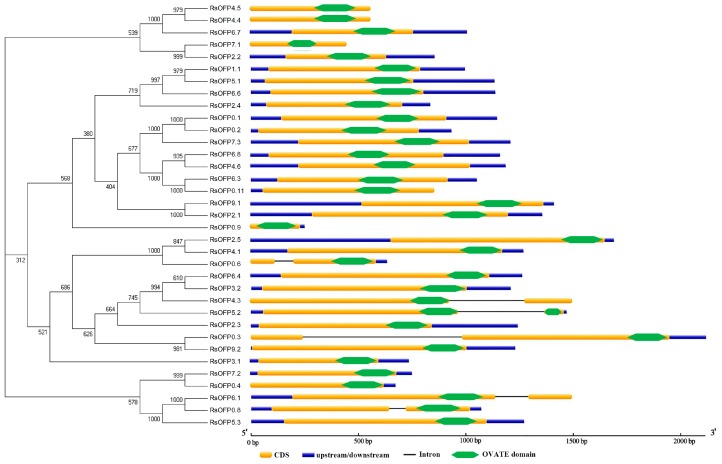
Phylogenetic analysis and genomic structures of *RsOFPs*. The phylogenetic tree displays only the topology. Yellow bars represent coding region sequences (CDS), dark blue bars represent upstream/downstream sequences, black lines represent introns, and diamond bars represent the OVATE domain.

**Figure 2:**
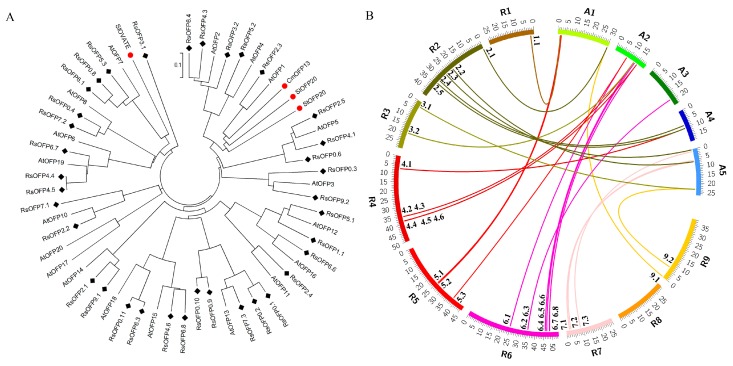
(**A**) Phylogeny of the ovate family proteins (*OFPs*) in radish, Arabidopsis, and other plant species in which mutations of *OFPs* have a discernable phenotypic effect on fruit or tuber shape, such as tomato, melon, and potato. *RsOFPs* are marked with black diamonds; SlOVATE, *SlOFP20*, *StOFP20*, and *CmOFP13* are marked with red dots. (**B**) Orthologous *OFP* genes between radish and Arabidopsis. Radish (R1–R9, Radish Genome Database) and Arabidopsis chromosome (A1–A5) maps were based on orthologous pair positions with Circos. Numbers on the chromosomes represent mega base pairs. The positions of *RsOFPs* on the chromosomes are indicated.

Thirty-five unique *RsOFPs* and five *RsOFP*-likes (with no/partial OVATE domain) were identified in the radish genome by BLASTP searches conducted in three different radish genome databases, with the known *AtOFPs* protein sequences as queries (Figure 1, Table S1). Analysis of exon-intron organization revealed that the majority of genes were intronless, while *RsOFP4.3*, *RsOFP5.2*, *RsOFP0.3*, *RsOFP0.6*, *RsOFP6.1*, and *RsOFP0.8* had one intron each (Figure 1). To provide a reference for the relationships among *OFP* proteins from radish and Arabidopsis, and functionally characterized *OFPs* from tomato, potato, and melon, a phylogenetic tree was constructed (Figure 2A). *RsOFP5.3*, *RsOFP0.8*, and *RsOFP6.1* clustered together with the tomato fruit shape gene SlOVATE and *AtOFP7*, while *RsOFP2.3* clustered together with *AtOFP1*, the potato tuber shape gene *StOFP20*, another tomato fruit shape gene *SlOFP20*, and the melon fruit shape gene *CmOFP13*. The *RsOFPs* were scattered throughout the nine chromosomes of radish except chromosome 8, and some chromosomes had a high density of *RsOFPs*. For instance, there were six *RsOFPs* on chromosome 6 (Figure 2B). Analysis of orthologous *OFP* genes between radish and Arabidopsis, taking the Radish Genome Database as an example, showed that *AtOFP18* had four orthologous radish genes, *AtOFP2* and *AtOFP6/19* had three orthologous radish genes, ten *AtOFPs* (*AtOFP3*, *AtOFP5*, *AtOFP7*, *AtOFP10*, *AtOFP12*, *AtOFP13*, *AtOFP14*, *AtOFP15*, *AtOFP17/AtOFP20*) had two orthologous radish genes, and five *AtOFPs* (*AtOFP1*, *AtOFP4*, *AtOFP8*, *AtOFP11*, *AtOFP16*) had only one orthologous radish gene (Figure 2B, Table S1).

### 2.2. Developmental Profiles of the Six Different Radish Cultivars

**Figure 3:**
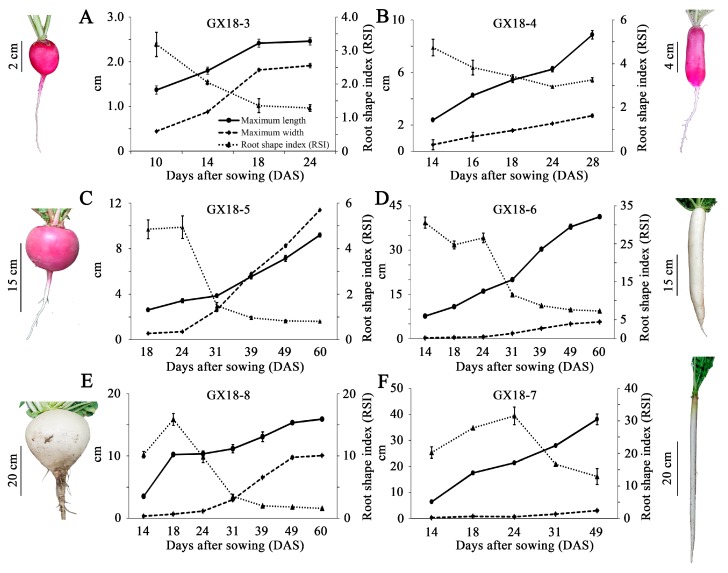
Growth parameters of six different radish cultivars during tuberous root development, including GX18-3 (**A**), GX18-4 (**B**), GX18-5 (**C**), GX18-6 (**D**), GX18-8 (**E**), and GX18-7 (**F**). Pictures of the radish cultivars are shown next to the plots. Error bars on each point indicate ±SE from at least three independent replicates.

Comparing gene expression profiles among different radish cultivars would be one of the easier ways to find the putative key genes involved in tuberous root shape formation. Thus, in this study, we chose six radish cultivars with different root shapes and sizes, including the famous cultivars Sakurajima-daikon (GX18-8) and Moriguchi-daikon (GX18-7), with the world’s largest (more than 20 kg) and longest (almost 2 m long) roots, respectively [3], and investigated their growth parameters. To characterize the root development patterns of these six different radish cultivars, the maximum length and width were measured periodically during the growth process of the tuberous root. Figure 3 and Figure S1 show that GX18-3, GX18-5, and GX18-8 had a root shape index (RSI) around 1, and these cultivars can be roughly classified as belonging to the round root shape group; while GX18-4, GX18-6, and GX18-7 had an RSI much higher than 1, and thus these cultivars were classified into the long root shape group. In the round root shape group, the maximum length and width of GX18-3 gradually increased at a similar rate from 10 to 18 days after sowing (DAS), with a very slow growth rate for the last six days (Figure 3A), while in GX18-5, the maximum length was higher than the maximum width before 39 DAS, after which the maximum width was higher, resulting in an oblate root shape (Figure 3C); the maximum length of GX18-8 increased significantly from 14 to 18 DAS, after which the maximum length and width increased at a similar rate (Figure 3E). In the long root shape group, the maximum length of the tuberous root gradually increased during tuberous root development, while the maximum width increased at a relatively slow rate, especially in GX18-6 and GX18-7, where the maximum width barely increased from 14 to 24 DAS (Figure 3B,D,F).

### 2.3. Expression Profiles of RsOFPs during Tuberous Root Development

**Figure 4:**
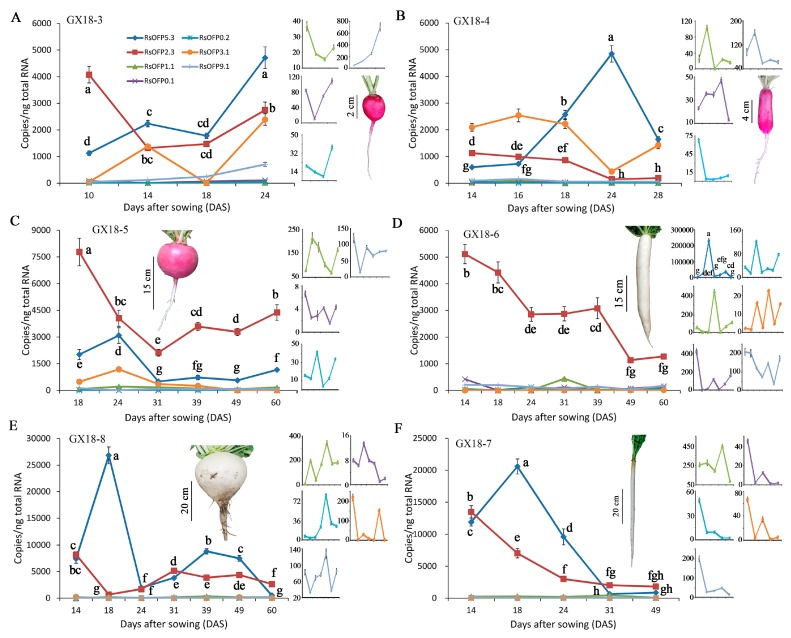
Expression profiles of seven *RsOFP* genes (*RsOFP5.3*, *RsOFP2.3*, *RsOFP1.1*, *RsOFP0.1*, *RsOFP0.2*, *RsOFP3.1*, *RsOFP9.1*) in six radish cultivars during tuberous root development, including GX18-3 (**A**), GX18-4 (**B**), GX18-5 (**C**), GX18-6 (**D**), GX18-8 (**E**), and GX18-7 (**F**). Genes with low expression are shown beside each chart. Error bars on each point indicate ±SE from at least three independent replicates. Different letters indicate significant differences as determined using ANOVA, followed by Tukey’s HSD test (*p* < 0.05).

To identify the putative *RsOFPs* involved in tuberous root shape formation in radish, the expression profiles of the *RsOFPs* were analyzed over time. Before conducting the expression analysis of the 35 putative *OFP* genes in our materials, a preliminary screening of their expression levels was carried out using data from the NODAI Radish Genome Database and Radish Genome Database. Genes that were barely expressed or had extremely low expression levels during the whole process of tuberous root development were omitted in our subsequent studies. Seven genes were selected for further analysis in our six radish cultivars (Figure 4). In GX18-3, GX18-4 and GX18-5, *RsOFP5.3*, *RsOFP2.3*, and *RsOFP3.1* were highly expressed while *RsOFP1.1*, *RsOFP0.1*, *RsOFP0.2*, and *RsOFP9.1* had relatively low expression levels. In GX18-6, GX18-7, and GX18-8, *RsOFP5.3* and *RsOFP2.3* were the two most highly expressed *RsOFPs*; the other five genes had relatively low expression during tuberous root development. Among the three genes with relatively high expression, *RsOFP5.3* and *RsOFP3.1* did not have a consistent expression pattern in either the round or long root shape groups, while *RsOFP2.3* had a similar expression pattern within the round root shape group cultivars and within the long root shape group cultivars. Expressions of *RsOFP2.3* first decreased and then increased in all three nearly round root shape cultivars, but gradually decreased in all three long root shape cultivars. It seemed that *RsOFP2.3* was the most likely candidate *RsOFP* gene involved in root shape formation in radish, as it not only showed a relatively high expression level but also exhibited different expression patterns between the round and the long root shape groups after the cortex splitting stage.

### 2.4. Overexpression of RsOFP2.3 in Arabidopsis Mainly Inhibited Organ Elongation

**Figure 5:**
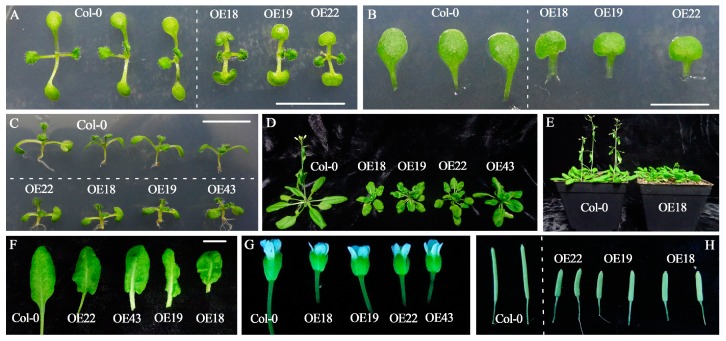
Phenotypic pictures of *RsOFP2.3*-overexpressing transgenic plants and the wild type (WT). (**A**) Top view of 7-day-old seedlings in the T2 transgenic lines OE18, OE19, and OE22 and the WT. Bar = 1cm. (**B**) Close-up view of cotyledons from 7-day-old seedlings of the T2 transgenic lines and WT. Bar = 0.5 cm. (**C**) Lateral view of 7-day-old seedlings of the T2 transgenic lines and WT for comparison of hypocotyls. Bar = 1 cm. (**D**) An overview of 26-day-old seedlings of the T2 transgenic lines and WT. (**E**) Bolting and flowering in transgenic compared with WT plants. Both were delayed in the transgenic plants. (**F**) Fully developed rosette leaves of the WT and transgenic lines. Bar = 1 cm. (**G**) Floral organs of the WT and transgenic lines. (**H**) Siliques of the WT and transgenic lines.

**Figure 6:**
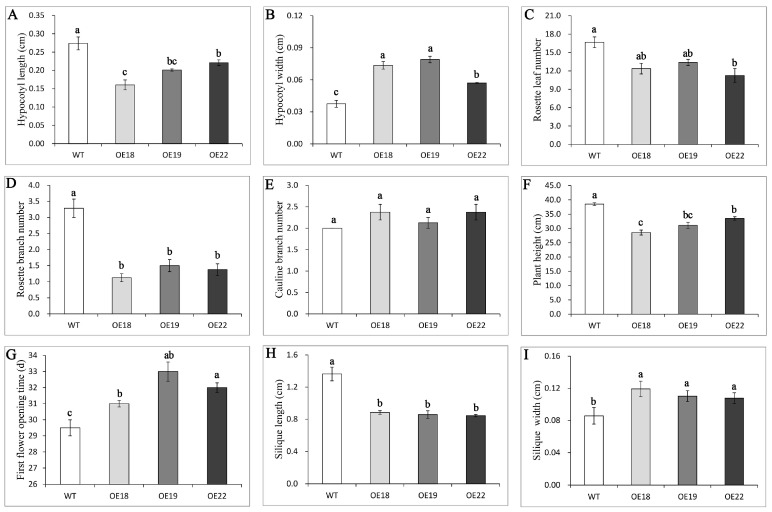
(**A**,**B**) Hypocotyl length and width of the T2 transgenic lines and WT in 7-day-old seedlings. (**C**,**D**,**E**) Rosette leaf number, rosette branch number, and cauline branch number in the transgenic and WT plants. (**F**) Plant height in the T2 transgenic lines and WT at 55 days after sowing. (**G**) First flower opening time in the T2 transgenic lines and WT. (**H**,**I**) Silique length and width in the T2 transgenic lines and WT. Error bars on each point indicate ±SE from at least five independent replicates. Different letters indicate significant differences as determined using ANOVA, followed by Tukey’s HSD test (*p* < 0.05).

To get further insight into the function of *RsOFP2.3*, it was ectopically expressed in Arabidopsis Col-0 plants driven by the 35S promoter. The independent hygromycin-resistant T1 transgenic plants were transferred to soil, grown in a growth chamber, PCR verified with specific primers for the 35S promoter and green fluorescent protein (GFP) (Table S2), and self-pollinated to obtain segregated T2 progeny. Three homozygous representative lines were selected for further analysis. Compared with the wild type, the overexpression lines showed significant phenotypic changes (Figure 5 and Figure 6). In terms of organ shape, the cotyledons were kidney-shaped, and the mature leaves were curled in the *RsOFP2.3*-overexpressing Arabidopsis lines (Figure 5A,B,F). Most interestingly, both hypocotyl and silique length were decreased, and their widths were increased (Figure 5C,H; Figure 6A,B,H,I). For organ size, the sizes of rosettes, leaves, and floral organs and plant height were all reduced (Figure 5D–G; Figure 6F). Moreover, in the transgenic lines, rosette branch number were significantly reduced while rosette leaf number and cauline branch number were not significantly changed (Figure 6C–E). In addition, the first flower opening time was found to be delayed (Figure 6G).

### 2.5. RsOFP2.3 Was Localized in the Cytoplasm

**Figure 7:**
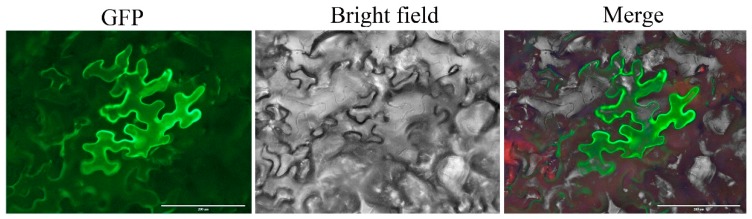
Subcellular localization of *RsOFP2.3*. The left column shows the green fluorescent protein (GFP) signal; the middle column shows the bright field image, and the right column shows the merged image.

To investigate the subcellular localization of the *RsOFP2.3* protein, a *p35S:RsOFP2.3-GFP* vector containing the open reading frame sequence of *RsOFP2.3* (without the termination codon) fused in-frame with the green fluorescent protein (GFP) and driven by the 35S promoter was constructed and transformed into leaf cells of N. benthamiana by Agrobacterium-mediated transient transformation. Our results showed that *RsOFP2.3* was localized in the cytoplasm (Figure 7), which was consistent with the subcellular localization of its ortholog SlOVATE in tomato.

### 2.6. RsOFP2.3 was Responsive to ABA, NAA, 6-BA, and GA_3_ Treatments

**Figure 8:**
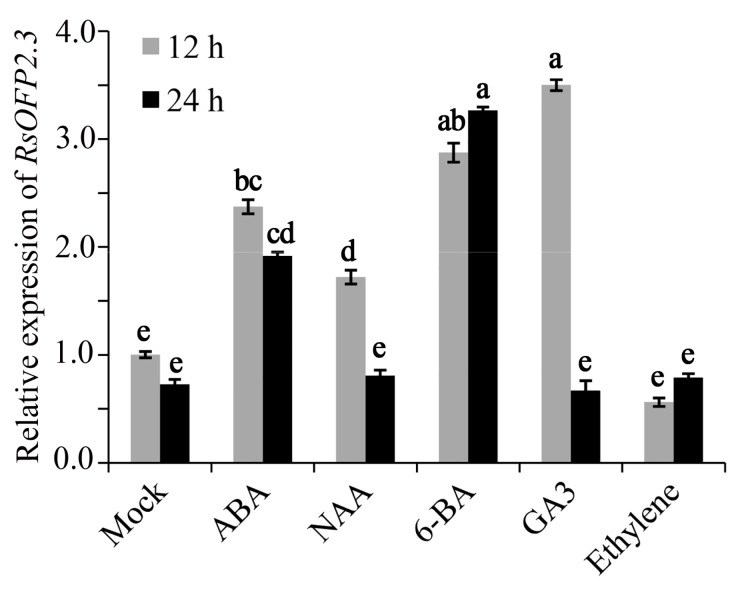
Responses of *RsOFP2.3* to five classic hormones in seedling roots after 12 h and 24 h treatment. Error bars on each point indicate ±SE from at least three independent replicates. Different letters indicate significant differences as determined using ANOVA, followed by Tukey’s HSD test (*p* < 0.05).

To further characterize *RsOFP2.3*, its responses to the five major plant hormones in the roots of young seedlings were investigated (Figure 8). Our results showed that expression of *RsOFP2.3* was significantly upregulated in response to abscisic acid (ABA), alpha-Naphthaleneacetic acid (NAA), 6-Benzylaminopurine (6-BA), and gibberellic acid (GA_3_) at 12 h, and was most sensitive to treatment with GA_3_, followed by 6-BA, ABA, and NAA, while there was no significant change in response to ethylene either at 12 h or 24 h after treatment. Moreover, *RsOFP2.3* expression was almost back to normal in the NAA and GA_3_ treatments after 24 h, while it was still significantly induced in ABA and 6-BA treatments.

## 3. Discussion

OVATE-like proteins have been identified in all land plants, including the early-diverged land plants *Physcomitrella patens* (moss) and *Selaginella moellendorffii* (spikemoss) [16]. Genome searches of sequenced land plants using the tomato OVATE protein revealed that all the plants examined contain *OFPs*, with 31 *OsOFPs* in the rice genome [33], 19 *AtOFPs* in the Arabidopsis genome [16], 19 *CsOFPs* in cucumber, 18 *CmOFPs* in melon, and 17 *ClOFPs* in watermelon [30]. In radish, 35 unique putative *RsOFPs* were identified from three radish genome databases. Previous studies have revealed that the common ancestor of *Brassica* and *Raphanus* experienced α’ whole-genome triplication event after its divergence from Arabidopsis. Consistent with this, there are more *OFPs* in radish than in Arabidopsis. However, gene losses of orthologous groups between *Arabidopsis*, *Brassica*, and *Raphanus* have taken place in both the *Brassica* and *Raphanus* lineages [34,35]. That is why the number of *OFPs* in radish was not three times more than that in Arabidopsis. The orthologous *OFP* gene pairs between Arabidopsis and radish, shown in Figure 2B and Table S1, also revealed gene losses of many orthologous groups. It was reported that overexpression of *AtOFP* genes with close phylogenetic relationships produced similar phenotypes [14], which indicates genes clustered together may have the same functions. It is of great interest that among the seven expressed *RsOFPs* in radish, only *RsOFP2.3* and *RsOFP5.3* were clustered together with fruit/tuber shape genes *CmOFP13*, *SlOFP20*, *StOFP20*, and SlOVATE (Figure 2A), indicating these two *RsOFPs* may have similar functions in regulating radish tuber shape formation.

To gain more insight into the role of *RsOFPs* in the regulation of tuberous root shape formation, the expression profiles of the seven expressed *RsOFPs* were detected in the six representative radish cultivars during root development (Figure 4). Overall, only *RsOFP2.3* showed a distinctive pattern between the long-type radish cultivars (GX18-4, GX18-6, GX18-7) and nearly round-type radish cultivars (GX18-3, GX18-5, GX18-8). *RsOFP2.3* expression was gradually downregulated during the development of all long-type radish cultivars, which was negatively correlated with increases in maximum length (Figure 3 and Figure 4). However, *RsOFP2.3* exhibited a different expression pattern in the nearly round radish cultivars, with decreased expression from the cortex splitting stage and then increased expression at the thickening stage (Figure 4). In GX18-5, the expression levels of *RsOFP2.3* were first downregulated from 18 to 31 DAS, when the maximum length was higher than the maximum width, and then increased from 39 to 60 DAS, at which time the maximum length was lower than the maximum width. Moreover, the expression pattern of *RsOFP2.3* in GX18-8 showed an opposite trend to that of maximum length during development. Overall, it seemed that *RsOFP2.3* expression was not well correlated with the increase in tuberous root width, while it was negatively related to the tuberous root elongation not only in the round radish group but also in the long radish group. These results made this gene the top candidate for the involvement of tuberous root shape. To further investigate the role of *RsOFP2.3* in organ shape formation, we generated *RsOFP2.3* overexpression lines in Arabidopsis. Our results showed that *RsOFP2.3* overexpression had multiple effects on Arabidopsis growth and development (Figure 5 and Figure 6), especially reducing the hypocotyl and silique lengths and also plant height, which was very similar to its homologous *AtOFPs*, especially *AtOFP1*, its closest homologous gene [13,14]. The radish tuberous root comprises two anatomically distinct parts, the upper part that originates from the hypocotyl and the lower part that consists of true root tissue [9]. The role of *RsOFP2.3* in reducing Arabidopsis hypocotyl length also supported its putative role in suppressing tuberous root elongation in radish. However, since the root in Arabidopsis does not enlarge as in radish and *RsOFP2.3*, overexpression in Arabidopsis did not lead to the inhibition of Arabidopsis root elongation, the reason why *RsOFP2.3* was negatively correlated with tuberous root elongation in radish may be more complex and needs to be further studied. It is noteworthy that the shape of tuberous root is already different at the cortex splitting stage (10 or 14 DAS); expression studies conducted as early as in the embryo or soon after germination would provide insightful information too. Moreover, as the radish has undergone artificial selection for root shape, including wild radish, it would provide an appropriate baseline control against which to compare the expression in these cultivars.

A previous study reported that when radish cultivars were grown under cytokinin-treated conditions for 1 week, their roots became noticeably thicker than the untreated roots [36]. This was consistent with our results that *RsOFP2.3* was induced by exogenous 6-BA, and overexpression of *RsOFP2.3* led to increased hypocotyl and silique widths (Figure 5, Figure 6 and Figure 8). Additionally, it was reported that *AtGA20ox1*, a gene encoding the key enzyme in GA (gibberellic acid) biosynthesis, is a target gene regulated by *AtOFP1*, and exogenous gibberellic acid can partially restore defects in cell elongation in plants overexpressing *AtOFP1* [13]. However, in radish, *RsOFP2.3* was significantly induced by exogenous GA_3_ treatment (Figure 8), indicating *RsOFP2.3* may have different mechanisms of suppressing root elongation rather than through the GA pathway. This was further supported by its different subcellular localization, as *AtOFP1* was reported to localize in the nucleus, while *RsOFP2.3* was found to localize in the cytoplasm ([13]; Figure 7), which was more similar to its homologous genes in tomato, such as SlOVATE [21]. In tomato, both *SlOFPs* and *SlTRMs* physically interact in plant cells, which leads to relocalization of one or the other protein, and then regulate cell division patterns in ovary development to alter final fruit shape [21]. Whether there are other proteins that interact with *RsOFP2.3* and lead to its relocalization and finally repressing the tuberous root elongation in radish needs to be further studied.

## 4. Materials and Methods

### 4.1. Plant Materials

Six different radish (*Raphanus sativus* L.) cultivars, namely Cherry belle (GX18-3), French Breakfast (GX18-4), Jinzhouwujinhong (GX18-5), Minowase (GX18-6), Moriguchi (GX18-7), and Sakurajima (GX18-8), were planted in an open field in August 2018 in the Beijing Vegetable Research Center (BVRC). GX18-3 and GX18-4 are European small radishes with 30-day growth periods, while GX18-5, 6, 7, and 8 are Asian big radishes with growth periods about 50 days or longer. The GX18-3, 5, and 8 cultivars can be classified into the group with a nearly round root shape, while the GX18-4, 6, and 7 cultivars have a relatively long cylindrical root. Roots at different developmental stages, including the initial cortex splitting stage and thickening stage, were periodically harvested and photographed according to the time points of developmental stages in the different varieties. For GX18-3, 10 days after sowing (DAS) was the cortex splitting stage, and 14–24 DAS was the thickening stage. For GX18-4, 14 DAS was the cortex splitting stage, and 16–28 DAS was the thickening stage. For GX18-5 and GX18-6, 18 DAS/14–18 DAS was the cortex splitting stage, and 24–60 DAS was the thickening stage. For GX18-7 and GX18-8, 14–18 DAS was cortex splitting stage, and 18–49 DAS/24–60 DAS was the thickening stage. According to the method described by Zaki et al. [6], each taproot was divided into three sections, top, middle, and bottom, by cutting the root transversely at two sites of equal distance along the vertical length. At least three independent biological replicates of taproot samples at each stage were collected. All samples were immediately frozen in liquid nitrogen and stored at −80 °C for subsequent analysis. The middle sections of the root were used for gene expression analysis.

### 4.2. Measurement of Growth Parameters in both Tuberous Root and Transgenic Arabidopsis

Measurement of the tuberous root and Arabidopsis silique and hypocotyl length and width were conducted using the ImageJ software (http://rsbweb.nih.gov/ij/). The root shape index is the ratio of root maximum length to maximum width. All parameters of transgenic Arabidopsis were from the T2 generation. Data were expressed as the average of at least five independent biological replicates.

### 4.3. Phytohormone Treatments

Seeds of Cherry belle with uniform size and plumpness were put into a 9 cm diameter petri dish lined with three pieces of filter paper and wetted with 4 ml deionized water. Three days after imbibition, the seeds were treated with deionized water or solutions including 100 μM abscisic acid (ABA, Sigma A1049), 100 μM 6-Benzylaminopurine (6-BA, Sigma B3274), 100 μM Gibberellic acid (GA_3_, Sigma G7645), 50 μM alpha-Naphthaleneacetic acid (NAA, Sigma N0640), and 50 μM Ethephon (Sigma 45473). At least 300 seeds were used for each treatment. Root samples were collected 12 and 24 h after treatment, immediately frozen in liquid nitrogen, and stored at −80 °C for further use.

### 4.4. Identification of Radish OFP Genes

The amino acid sequences of *AtOFPs* identified from previous studies were obtained from the Arabidopsis Information Resource (https://www.arabidopsis.org/index.jsp). The protein sequences of *OFPs* (SlOVATE, *SlOFP20*, *StOFP20*, and *CmOFP13*) with a discernable phenotypic effect on fruit or tuber shape were obtained from the Sol Genomics Network (https://solgenomics.net/) and the Cucurbit Genomics Database (http://cucurbitgenomics.org/), respectively. To identify all possible radish *OFP* genes, BLASTP searches were conducted in different radish genome databases, including the NODAI Radish genome database (http://www.nodai-genome-d.org/), Radish Genome Database (http://radish-genome.org/), and *Raphanus sativus* Genome Database (http://radish.kazusa.or.jp/), with the above mentioned *AtOFP* protein sequences as queries. The putative *RsOFPs* were designated according to their chromosomal locations in the Radish Genome Database (chromosome information was not available from the other two radish genome databases); for example, the first *OFP* gene on chromosome 1 was named *RsOFP1.1*. *OFP* genes that had no chromosome information were designated as being on chromosome 0.

### 4.5. Structure and Phylogenetic Analysis of the OFP Genes

The exon/intron structures of the radish *OFP* genes were analyzed with the online tool Gene Structure Display Server (http://gsds.cbi.pku.edu.cn) [37]. The OVATE domain was identified using the Conserved Domain Search Service (CD Search) from the National Center for Biotechnology Information (https://www.ncbi.nlm.nih.gov/Structure/cdd/wrpsb.cgi). Phylogenetic trees were constructed using the Neighbor-Joining (N-J) method in the ClustalX software with the bootstrap analysis setting at 1000 replicates to evaluate the reliability of different phylogenetic groups. Tree files were visualized and edited using the MEGA 4.0.2 software. The relationships of orthologous genes between radish and Arabidopsis were plotted using the Circos software.

### 4.6. RNA Extraction, RT-PCR, and Real-time PCR Analysis

Total RNA was extracted using the Huayueyang Quick RNA isolation Kit (Cat. No.: ZH120, Huayueyang Biotechnology, Beijing, China) following the manufacturer’s instructions. The quantity and quality of the total RNA were checked using a NanoDrop 1000 spectrophotometer (Thermo Fisher Scientific Inc.; Waltham, MA, USA) and by resolution on a 1% non-denaturing agarose gel, respectively. cDNA was synthesized from the total extracted RNA using the FastKing RT Kit (with gDNase) (Tiangen Biotech, Beijing, China) according to the manufacturer’s instructions. qRT-PCR assays were performed using the LightCycler480 RT-PCR system (Roche, Switzerland) with specific primers (Supplementary Table S2). Each reaction consisted of 10 μL SYBR Green I Master Mix, 5 μL cDNA (20 ng/μL) and 5 μL primer mix (2 μM of each primer) to make a total volume of 20 μL. Reactions were carried out under the following conditions: 95 °C for 5 min, followed by 40 cycles of 95 °C for 20 s, 60 °C for 20 s, and 72 °C for 20 s. PCR amplification of a single product of the correct size for each gene was confirmed by agarose gel electrophoresis and sequencing. RNA polymerase-II transcription factor (RPII) was used to standardize each reaction run with respect to RNA integrity, sample loading, and inter-PCR variations [38].

### 4.7. Subcellular Localization and Generation of RsOFP2.3-Overexpression Transgenic Lines

To determine the subcellular localization of *RsOFP2.3*, the open reading frame sequence of *RsOFP2.3* (without the termination codon) was subcloned into the pSuper1300-GFP vector to generate the *35S:RsOFP2.3-GFP* construct. The specific primers used are listed in Supplementary Table S2. Four- to 5-week-old leaves of N. benthamiana seedlings were selected, and Agrobacterium carrying *p35S:RsOFP2.3-GFP* was injected into the leaves. The system was subsequently cultured in the dark for 1 day and then under normal illumination for another 1–2 days under 25 °C. Finally, the infiltrated leaves were examined, and GFP signals from the infiltrated hypodermis of the tobacco leaves were observed under a Leica fluorescence microscope (Leica Microsystem, Heidelberg, Germany). To generate the *RsOFP2.3*-overexpression transgenic lines, Agrobacterium tumefaciens strain GV3101 carrying *p35S:RsOFP2.3-GFP* was transformed into Arabidopsis Col-0 plants using the floral dip method [39].

## 5. Conclusions

In this study, we identified 35 putative *RsOFP* genes from radish, analyzed their gene structures and phylogeny, and investigated their possible roles in the regulation of tuberous root shape. Our results showed that *RsOFP2.3* may suppress root elongation and was the most likely candidate involved in tuberous root shape. Moreover, the ectopic expression of *RsOFP2.3* in Arabidopsis further revealed it has multiple functions in organ shape formation.

## Supplementary Materials

Supplementary Materials can be found at https://www.mdpi.com/1422-0067/21/4/1293/s1. Figure S1. Root shape indexes of GX18-3, GX18-4, GX18-5, GX18-6, GX18-7, and GX18-8. Table S1. Information on the 40 putative *RsOFPs*. Table S2. Primers used in this study.

## Abbreviations

*OFPs*	OVATE FAMILY PROTEINS
QTL	Quantitative Trait Locus
DUF623	Domain of Unknown Function 623
TRM	TONNEAU1 RECRUITMENT MOTIF
TTP	TON1-TRM-PP2A
DAS	Days after sowing
CDS	Coding region sequences
RSI	Root Shape Index
GFP	Green fluorescent protein
ABA	Abscisic acid
6-BA	6-Benzylaminopurine
GA3	Gibberellic acid
NAA	Alpha-Naphthaleneacetic acid
