# Supplementary material for: Characterization of the OFP Gene Family and its Putative Involvement of Tuberous Root Shape in Radish
Source: Int J Mol Sci. 2020 Feb 14;21(4):1293. doi: 10.3390/ijms21041293 (PMC7072887; doi:10.3390/ijms21041293)
Supplement: Supplementary file 1 [file ijms-21-01293-s001.zip › Supplementary/Table S2.docx]

Table S2 Primers used in this study.

| Gene | Forward primer | Reverse primer |
| --- | --- | --- |
| Real time PCR primers | | |
| *RsOFP5.3* | AGGATCCGTACGAAGATTTCAAAGG | AATCGGAGAGTTGAACACTGGACCT |
| *RsOFP2.3* | AGCAGAGCGGTAGTGAAGGCGT | TGGGGTGAAAGATTAAGATCAAGCC |
| *RsOFP1.1* | GAGGAGCCGCCATGAAGCAAT | TTAGTCCGGTGACCGTCCGATAAG |
| *RsOFP0.1* | AGAGCTTGGAGTCGATGCTCGAGT | GACGATAACGAATATAACGGCGACG |
| *RsOFP0.2* | TGGTATTTGAGGATGAACGAGAGGA | TTAGACGATAACGAACATAACGGCG |
| *RsOFP3.1* | AGTGATGATGGCAATGGAGAAATGT | TCGTAAAAGATCTCGAGAATGGCAG |
| *RsOFP9.1* | GGAGCGAGGTGGACTGGGACT | CCATCCTCTTCCTAAGCCTCTCCC |
| *RPII* | CTCTCTGGGGCTTTAGCCATCTTCT | GGTCATTGTCCTGACGGTTCTGAAT |
| Primers used for *35S:RsOFP2.3-GFP* construction | | |
| *RsOFP2.3* | AAAACTGCAGATGGGTAATTATCGGTTTAAGCTAT | CGGGGTACCAAAAGAAAGATGGGGTGAAAGATTA |
| Primers used for verification of *35S:RsOFP2.3-GFP* transgenic lines | | |
| 35S-GFP | CGCGGGTATTCTGTTTCTATTCCAA | CAGCTCCTCGCCCTTGCTCACCAT |
