# Supplementary figures and images for: Characterization of the OFP Gene Family and its Putative Involvement of Tuberous Root Shape in Radish
Source: Int J Mol Sci. 2020 Feb 14;21(4):1293. doi: 10.3390/ijms21041293 (PMC7072887; doi:10.3390/ijms21041293)

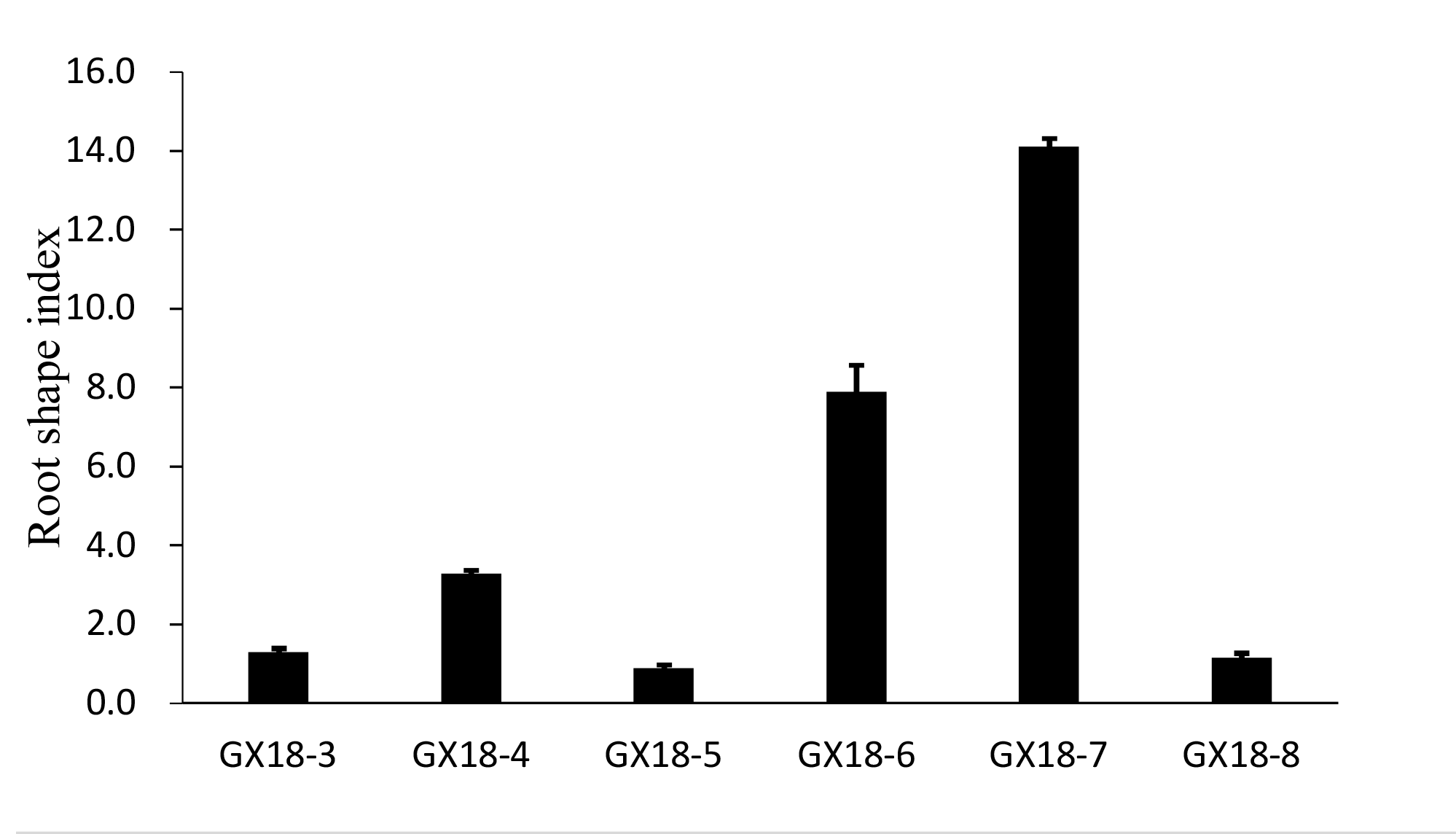

Supplement: Supplementary file 1 [file ijms-21-01293-s001.zip › Supplementary/Fig S1.tif]
